# Gd-Complex of a Rosmarinic Acid Conjugate as an Anti-Inflammatory Theranostic Agent via Reactive Oxygen Species Scavenging

**DOI:** 10.3390/antiox9080744

**Published:** 2020-08-13

**Authors:** Hee-Kyung Kim, Seonghwan Hwang, Bokyung Sung, Yeoun-Hee Kim, Yongmin Chang

**Affiliations:** 1 Laboratory Animal Center, Daegu-Gyeongbuk Medical Innovation Foundation, Dong-gu, Daegu 41061, Korea; hkkim@dgmif.re.kr; 2 Department of Medical & Biological Engineering, Kyungpook National University, Daegu 41944, Korea; tjdghks4277@naver.com (S.H.); xxgzxz4@naver.com (B.S.); 3 Mirae BioPharm. Co., 124, Sagimakgol-ro, Jungwon-gu, Gyeonggi-do 13207, Korea; bigeye38@naver.com; 4 Department of Radiology, Kyungpook National University Hospital, Daegu 41944, Korea; 5 Department of Molecular Medicine, School of Medicine, Kyungpook National University, Daegu 41944, Korea

**Keywords:** rosmarinic acid, Gd-chelate, anti-inflammatory effects, antioxidants, theranostic agent

## Abstract

Rosmarinic acid (RosA), an important polyphenol, is known for its antioxidant and anti-inflammatory activities. However, its application in theranostics has been rarely reported. Therefore, a new single-molecule anti-inflammatory theranostic compound containing RosA would be of great interest. A gadolinium (Gd) complex of 1,4,7,10-tetraazacyclododecane-1,4,7-trisacetic acid (DO3A) and RosA (Gd(DO3A-RosA)(H_2_O)) was synthesized and examined for use as a single-molecule theranostic agent. Its kinetic stability is comparable to that of clinically used macrocyclic magnetic resonance imaging contrast agents. In addition, its relaxivity is higher than that of structurally analogous Gd-BT-DO3A. This agent was evaluated for inflammatory targeting magnetic resonance contrast and showed strong and prolonged enhancement of imaging in inflamed tissues of mice. The theranostic agent also possesses antioxidant and anti-inflammatory activities, as evidenced by reactive oxygen species scavenging, superoxide dismutase activity, and inflammatory factors. The novel RosA-conjugated Gd complex is a promising theranostic agent for the imaging of inflamed tissues, as well as for the treatment of inflammation and oxidative stress.

## 1. Introduction

Rosmarinic acid (RosA) is an important polyphenol that exhibits several pharmacological properties, including antioxidant, antiallergic, oxidative stress inhibition, anti-inflammatory, anticancer, and immunomodulatory activities [1,2,3,4,5]. It is an ester of caffeic acid and 3,4-dihydroxyphenyllactic acid and is commonly found in herbal plants, such as rosemary, sage, and mint [1]. Considerable efforts have been expended on developing various RosA derivatives as effective antioxidant and anti-inflammatory agents [2,3,4]. For example, RosA complexed with triphenyl phosphonium permeates mitochondrial membranes and scavenges reactive oxygen species (ROS) [6]. The synthesis of various RosA derivatives based on structure–activity relationships may yield an antioxidant agent that prevents amyloid-β aggregation [7]. The acetyl ester derivative of RosA has been proposed as an anti-inflammatory agent with therapeutic applications [8].

Inflammation is a component of many common diseases and is a vital immune response to various factors, including proinflammatory cytokines, such as interleukin-1 (IL-1) and tumor necrosis factor (TNF) [9]. The overproduction of free radicals associated with the inflammatory process can generate oxidative stress and damage biomacromolecules [10]. In particular, ROS can play an important role in inflammatory disease [11]. Increased intracellular ROS levels induce the synthesis and activation of cyclooxygenase-2 (COX-2), a key molecule in the biosynthesis of prostaglandins, and are important inflammatory mediators in human disease [12,13]. Therefore, agents that reduce ROS levels can be valuable for the treatment of inflammation triggered by oxidative stress.

Despite notable antioxidant and anti-inflammatory effects, RosA is rarely indicated for use in theranostics (simultaneous therapy and diagnosis). Two nanoplatforms are currently available. First, RosA can be encapsulated in lipid- or polymer-based nanoparticles for protection against amyloid-β insult or for anticancer effects and allows simultaneous fluorescence imaging [14,15]. More recently, RosA loaded onto nanosized metal-organic frameworks (MOFs) has been studied for combined anticancer therapy and fluorescence imaging [16]. There is a significant demand for theranostic agents that can use RosA for imaging not dependent on fluorescence. Fluorescence imaging has limited clinical application due to the lack of penetration of the imaging signal. Therefore, the design of novel single-molecule-based anti-inflammatory theranostic compounds including RosA is of great interest in medicinal chemistry.

In this study, a novel theranostic agent incorporating gadolinium and RosA, called GdL (3), was synthesized as a single molecule. This agent combines a magnetic resonance-imaging (MRI) agent (gadolinium or Gd) with an anti-inflammatory drug (RosA) and enables the diagnosis of inflamed tissue via MRI. The antioxidant and anti-inflammatory activities of this agent exploited the scavenging effects on 2,2-diphenyl-1-picrylhydrazyl (DPPH), the inhibition of COX-2 production, and suppression of the proinflammatory cytokine TNF-α. In particular, this novel agent was evaluated to determine whether its modified structure compromised the anti-inflammatory activity of the parent compound. This new complex is a rare example of a small-molecule theranostic agent-based RosA derivative.

## 2. Materials and Methods

### 2.1. Materials

RosA, 1-hydroxybenzotriazole hydrate (HOBt), and gadolinium (III) acetate hydrate were purchased from Sigma Aldrich (St. Louis, MO, USA). Other reagents and solvents were purchased from Tokyo Chemical Industry Co., Ltd. (Tokyo, Japan) and Duksan Pure Chemicals Co., Ltd. (Ansan-si, Gyeonggi-do, Republic of Korea). Tri-*tert*-butyl 2,2′,2″-(10-(2-([2-aminoethyl]amino)-2-oxoethyl)-1,4,7,10-tetraazacyclododecane-1,4,7-triyl)triacetate (DO3A-*^t^*Bu-NH_2_) was prepared according to a previously described method [17]. All chemicals were of analytical grade.

### 2.2. Instruments

Deionized water was used for all experiments. Proton nuclear magnetic resonance (^1^H NMR) experiments were performed on a Bruker Advance 500 spectrometer. Chemical shifts were given as d values with reference to tetramethylsilane (TMS) as an internal standard. Coupling constants were given in Hz. High-resolution fast atom bombardment mass (FAB mass) spectra were obtained from the Korean Basic Science Institute using a JMS-700 model (Jeol, Japan) mass spectrometer. A preparative high-pressure liquid chromatography (prep-HPLC; LC/Forte/R, YMC, Japan) system equipped with a YMC-Actus Triart C_18_ column (250 × 20.0 mm. inner diameter, S-5 µm, 12 nm, YMC, Japan) was used for purification. The prep-HPLC system used ultraviolet–visible (UV–vis) detection at 330 and 365 nm. The purity of all products was determined to be above 95% by the HPLC spectra. A flash column chromatography system (Isolera Prime, Biotage, Uppsala, Sweden) with SNAP KP-C_18_-HS 12 g was used for purification. The system used UV–vis detection at 254 and 330 nm. Gd concentration data used to confirm lipophilicity were measured using the Optima 7300DV and Avio 500 inductively coupled plasma (ICP) spectrometers (Perkin Elmer, Waltham, MA, USA). UV–vis absorption and fluorescence measurements were performed with a SpectraMax^®^ i3 (Molecular Devices, San Jose, CA, USA) using 96-well cell culture plates at 25 °C.

### 2.3. Synthesis and Characterization

Tri-tert-butyl 2,2′,2″-(10-(2-[(2-(3-(3,4-dihydroxyphenyl)-2-((3-(3,4-dihydroxyphenyl)acryloyl)oxy)propanamido)ethyl]amino)-2-oxoethyl)-1,4,7,10-tetraazacyclododecane-1,4,7-triyl)(R,E)-triacetate (**1**). A solution of 1-ethyl-3-(3-dimethylaminopropyl) carbodiimide (EDC) hydrochloride (0.3831 g, 1.9987 mmol) and HOBt (0.270 g, 1.9987 mmol) in dimethylfumarate (DMF, 0.8 mL) was added dropwise to a stirred suspension of RosA (0.655 g, 1.8179 mmol) and DO3A-^t^Bu-NH_2_ (0.117 g, 1.8179 mmol) in DMF (3 mL) at 0°C, and the mixture was stirred for 30 min. N,N-Diisopropylethylamine (DIPEA, 0.633 mL, 3.6358 mmol) was added dropwise to the mixture, which was stirred at room temperature for 17 h. The mixture was precipitated with cold water. The solid was isolated using filtration, washed several times with water, and dried under vacuum. Further purification was accomplished via flash column chromatography (C_18_ column; flow rate, 12 mL/min; trifluoroacetic acid (TFA) buffer; t_r_, 12 min 44 s) in a water (A)/acetonitrile (B) gradient as follows: from 5% B to 38% B for 10 min, isocratic elution with 38% B for 5 min, increase from 38% B to 100% for 1 min, and isocratic elution with 100% acetonitrile B for 4 min. The product was obtained as a yellow solid after drying under vacuum. Yield: 1.21 g (69.5%). ^1^H-NMR (500 MHz, DMSO-d_6_) δ (ppm) 7.91 (d, J = 8.4 Hz, 1H, -CHAr), 7.47–7.41 (m, 2H, ArCH_2_-), 7.34 (t, 1H, -COCHO-), 7.06 (s, J = 1.9 Hz, 1H, -Ar-), 6.98 (d, J = 8.3 Hz, 1H, -Ar-), 6.79 (d, J = 8.1 Hz, 1H, -Ar-), 6.67 (s, J = 1.9 Hz, 1H, -Ar-), 6.63 (d, J = 8.0 Hz, 1H, -Ar-), 6.50 (d, J = 8.0 Hz, 1H, -Ar-), 6.24 (d, J = 15.9 Hz, 1H, -COCH-), 3.76–2.61 (m, 28H, in the cyclen ring), 1.43 (d, J = 9.1 Hz, 27H, -CH_3_); HR-FAB-mass (*m*/*z*): Calculated for C_48_H_72_N_6_O_14_, 957.5185 ((MH)^+^); Found, 957.5181 ((MH)^+^).

(R,E)-2,2′,2″-(10-(2-[(2-(3-(3,4-dihydroxyphenyl)-2-((3-(3,4-dihydroxyphenyl)acryloyl)oxy)propanamido)ethyl)amino]-2-oxoethyl)-1,4,7,10-tetraazacyclododecane-1,4,7-triyl)triacetic acid (**2**). TFA (7.25 mL, 94.8 mmol) was added to (**1**) (1.21 g, 1.264 mmol), and the solution was stirred for 18 h at room temperature for deprotection of the tert-butyl moiety. After 18 h, TFA was evaporated, and the solution was repeatedly washed with methylene chloride and methanol to remove residual TFA. The crude brown solid was dissolved in methanol and precipitated with cold diethyl ether. The yellow solid was isolated using filtration, washed several times with diethyl ether, and dried under vacuum. The crude compound was purified via flash column chromatography (C_18_ column; flow rate, 12 mL/min; t_r_, 12 min 42 s) with a water (A)/acetonitrile (B) gradient as follows: 5% solvent B at 0 min to 21% solvent B in 13 min, isocratic elution with 21% solvent B for 2 min 40 s, increase to 40% solvent B in 15 min 40 s and then to 100% solvent B in 3 min 15 s, and isocratic elution with 100% solvent B for 4 min. The product was obtained as a yellow solid after drying under vacuum. Yield: 0.7 g (70.2%). ^1^H-NMR (500 MHz, D_2_O) δ (ppm) 7.36 (d, J = 15.9 Hz, 1H, -CH_2_Ar), 6.94 (s, 1H, -Ar-), 6.84 (d, J = 7.6 Hz, 1H, -Ar-), 6.73 (d,d,s, J = 19.8, 11.2 Hz, 3H, -Ar-), 6.59 (d, J = 6.9 Hz, 1H, -Ar-), 6.13 (d, J = 15.9 Hz, 1H, -COCHCH-), 5.06 (t, J = 6.2 Hz, 1H, -COCHC-), 3.75–2.75 (m, 30H, in the cyclen ring, ArCH_2_-, -NCH_2_CH_2_N-); HR-FAB-mass (*m*/*z*): Calculated for C_36_H_49_N_6_O_14_, 789.3307 ((MH)^+^); Found: 789.3306 ((MH)^+^); Anal. Calculated for C_36_H_49_N_6_O_14_·4or Cl_2_O: C, 46.43; H, 5.06; N, 8.12; O, 29.37; Found, C, 46.59; H, 4.89; N, 8.26.

GdL (3). Compound (**2**) (0.7 g, 0.887 mmol) was suspended in methanol, and Gd (III) acetate hydrate (0.296 g, 0.887 mmol) was added to the solution. The reaction mixture was stirred at 60°C for 22 h. The ivory-colored solution was removed under reduced pressure, and the crude compound was purified via flash column chromatography (C_18_ column; flow rate, 12 mL/min; t_r_, 32 min 20 s) using a water/acetonitrile gradient as follows: 5% solvent B at 0 min, isocratic elution with 5% B for 20 min, increase to 28% B in 40 min and then to 100% B in 2 min, and isocratic elution with 100% B for 10 min. The yellow solid product was obtained by drying under vacuum. Yield: 0.17 g (19.9%). HR-FAB-mass (*m*/*z*): Calculated for C_36_H_46_GdN_6_O_14_: 944.2313 ((MH)^+^); Found: 944.2319 ((MH)^+^).

### 2.4. Relaxivity

Relaxivity samples of gadolinium contrast agents were prepared in phosphate-buffered saline (PBS) at various concentrations (0.0625–1 mM). Additionally, human serum albumin (HSA) was dissolved in water (0.67 mM) to measure relaxivity in the presence of serum protein. The Signa Architect 3.0 T System (127.8 MHz, GE Healthcare, Milwaukee, WI, USA) was used for the experiment. *T*_1_ measurement was performed using the inversion recovery method, with various inversion times (TI) ranging from 50 to 1800 ms. *T*_1_ relaxation times were calculated from the nonlinear least-squares fit of the signal intensity measured at each TI value. *T*_2_ measurement was performed using spin-echo (SE) measurement with a Carr-Purcell-Meiboon-Grill pulse sequence. Fifteen MR images were acquired using 15 echo times (TE) ranging from 8.5 to 135 ms. *T*_2_ relaxation times were calculated from the nonlinear least-squares fit of the signal intensity measured at each TE. Relaxivity (*R*_1_ and *R*_2_) was calculated using a linear fit at each relaxation rate and concentration of the GdL (3) solution (1, 0.5, 0.25, 0.125, and 0.0625 mM). Relaxation times (*T*_1_ and *T*_2_) and relaxivity (*r*_1_ and *r*_2_) were image-processed to create maps of relaxivity and relaxation time.

### 2.5. Lipophilicity

Lipophilicity measurements were performed using a previously described method [18]. GdL (3) was dissolved in water to a volume of 1 mL at a concentration of 1 mM and mixed with the same volume of 1-octanol. The mixture was shaken for 48 h and was then allowed to stand at room temperature for 24 h. The obtained water and octanol phases were then separated, and the separated layers were pretreated with hydrochloric acid (~10 mL) and nitric acid (~10mL) at high temperatures to measure the Gd concentration. The Gd concentration in each layer was confirmed via ICP mass spectrometry (MS). Octanol–water partition coefficients were obtained from Equation (1) [18], where log *P* is the common logarithm of the partition coefficient, and *C_o_* and *C_w_* are the concentrations of Gd in the 1-octanol and water layers, respectively.

(1) $$\log P=\log(\frac{C_{o}}{C_{w}})$$

### 2.6. Transmetalation Kinetics

Transmetalation kinetics were measured using a previously described method [19]. Briefly, the protocol involved the evaluation of the water proton relaxation rate (*R*_1_^P^). A phosphate-buffered solution (pH 7.4) containing equimolar amounts of gadolinium complex and zinc chloride was used. A 10-μL ZnCl_2_ solution (100 mM) was added to 1 mL of the paramagnetic complex solution (1 mM). The mixture was vigorously stirred and immediately measured for 72 h. Control studies were performing using Gd-BT-DO3A (Gadovist^®^), Gd-DTPA-EOB (Primovist^®^), Gd-DTPA-BOPTA (MultiHance^®^), Gd-DTPA (Magnevist^®^), and Gd-DOTA (Dotarem^®^), with ZnCl_2_ for comparison. *R*_1_ relaxation rate is the reciprocal of the observed relaxation time, *T*_1_. The relative value of *R*_1_^P^ at any time t, *R*_1_^P^(t)/*R*_1_^P^(0), was represented as an estimate of the extent of transmetalation. Measurements were performed using the Signa Architect 3.0 T System (127.8 MHz, GE Healthcare, Milwaukee, WI, USA) at 293 K. Values are expressed as mean ± SD (n = 3).

### 2.7. Animal Models

Animal models for inflammation studies were developed following the guidelines of the Institutional Animal Care Use Committee of Kyungpook National University (2019-0129). Twenty-eight 6-weeks-old male institute of cancer research (ICR) mice (weight, 20–22 g; purchased from DBL, Eumseong, Korea) were used in this study. Mice were housed in a 12-h dark/light cycle with ad libitum access to water and food. Mice were anesthetized with 1.5–2.0% isoflurane in oxygen and received an intramuscular injection of turpentine oil (100 µL) in the left thigh muscle to induce inflammation. Turpentine oil was purchased from Daejung Chemicals & Metals Co., Ltd. (Korea). Three days after turpentine treatment, mice were used for in vivo MRI experiments [20]. For the in vivo anti-inflammatory study, mice were randomly divided into four groups: normal (n = 5), turpentine oil (n = 8), turpentine oil and GdL (3) (n = 8), and turpentine oil and RosA (n = 7). GdL (3) (0.1 mmol Gd/kg) and RosA (0.1 mmol/kg) were intravenously administered on day 4 after turpentine oil inoculation. At 24 h after drug injection, mice were euthanized, and the muscle tissue was extracted.

### 2.8. In Vivo MRI

In vivo MRI study was performed using the Signa Architect 3.0 T System (127.8 MHz, GE Healthcare, Milwaukee, WI, USA) equipped with a hand-wrist radiofrequency (RF) coil. The coil was a receiver type with an inner diameter of 80 mm. Seven- to approximately eleven-week-old male BALB/c-nu mice weighing 24–28 g and 6-week-old ICR mice weighing 25–28 g were used. The mice were anesthetized with 1.5–2.0% isoflurane in oxygen. GdL (3) and Gd-BT-DO3A at a concentration of 0.1-mmol Gd/kg body weight were injected into the tail vein. MRI measurements were performed before and after injection.

The coronal imaging parameters for SE of human xenograft models were as follows: repetition time (TR) = 450, TE = 8.8, field of view (FOV) = 8 mm, 256 × 192 matrix size, 1.2 slice thickness, numbers of acquisition (NEX) = 4, spacing = 0.1, and scan time = 3 min 11 s. For axial images, parameters were as follows: TR = 450 ms, TE = 8.8 ms, 8 mm FOV, 192 × 128 matrix size, 1.5 slice thickness, NEX = 2, spacing = 0.3, and scan time of each image: 1 min 43 s. The contrast-enhanced anatomical locations were identified as the heart, liver, gallbladder, kidneys, and inflamed tissues. For quantitative measurement, signal intensities in particular regions of interest were measured using the Advantage Window software (GE Medical, USA). Contrast-to-noise ratio (CNR) was obtained using the following equation, where SNR is the signal-to-noise ratio (2):

(2) $${\mathrm{CNR}=\mathrm{SNR}}_{\mathrm{post}}\;{-\;\mathrm{SNR}}_{\mathrm{pre}}$$

### 2.9. Cell Culture

Seven- to approximately 11-week-old male BALB/c-nu mice weighing 24–28 g and 6-week-old ICR C2C12 mouse myoblast cells (ATCC^®^CRL-1772) were cultured from four to nine passages to 70–80% confluency in Dulbecco’s modified Eagle’s medium (DMEM, WelGENE, Daegu, Korea) supplemented with 10% (*v*/*v*) fetal bovine serum and 1% antibiotics-antimycotics (Gibco, USA). Cells were incubated in a humidified 5% CO_2_ atmosphere at 37 °C.

To induce cell differentiation, cells were seeded and incubated with DMEM containing 5% (*v*/*v*) horse serum (Gibco, USA) and 1% antibiotic-antimycotics (Gibco, USA) to initiate the differentiation of myoblasts into myotubes. The medium was replaced every 1 to 2 days.

### 2.10. Cell Viability

To evaluate viability, undifferentiated cells were seeded into a 96-well plate (0.5 × 10^2^ cells/well) and incubated in the culture medium until stabilization. The medium was then changed to a differentiation medium, and the cells were treated with or without drugs (0~400 μM range of ascorbic acid (AA), Gd-BT-DO3A, RosA, and GdL (3)) after 3 days. Cells were incubated for 24 h with the drugs. The Cell Counting Kit-8 (CCK-8, Dojindo Laboratories, Kumamoto, Japan) solution was then added to each well, and the plate was incubated for 2 h. Absorbance was measured at 450 nm using a microplate reader (SpectraMax i3, Molecular Devices, CA, USA). The experiment was performed three times.

### 2.11. RNA Isolation and Quantitative Real-Time Polymerase Chain Reaction (RT-PCR)

Total RNA was isolated from mouse thigh muscle tissue/C2C12 mouse muscle cells using the Trizol reagent (Life Technologies, Rockville, MD, USA) following the manufacturer’s instructions. One microgram of total RNA was reverse-transcribed into complementary DNA using the AccuPower CycleScript RT PreMix (Bioneer, Daejeon, Korea). Real-time PCR was performed using the Power SYBR Green Premix (Cat no. 4367659, Thermo Fisher Scientific, Waltham, MA, USA) and Quantstudio 3 Real-time PCR system (Thermo Fisher Scientific, USA) according to the manufacturer’s protocol. The amplification conditions were as follows: hold stage: one cycle at 95 °C for 10 min; PCR stage: 40 cycles at 95 °C for 15 s and at 60 °C for 40 s; and melt curve stage: 95 °C for 15 s, 60 °C for 60 s, and 95 °C for 15 s. Relative quantification of mRNA expression was performed using the Quantstudio™ Design & Analysis Software. The following specific oligonucleotide primer sequences were used to amplify different segments: SOD1: 137 bp, 5′-GCCCGGCGGATGAAG-3′ and 5′-CACCATTGTACGGCCAATGATG-3′, COX-2: 99 bp, 5′-GAACCTGCAGTTTGCTGTGGG-3′ and 5′-TCGCACACTCTGTTGTGCTCC-3′, TNF-α: 105 bp, 5′-GGTTCTGTCCCTTTCACTCA-3′ and 5′-CCTCTTCTGCCAGTTCCA-3′, and GAPDH: 85 bp, 5′-CTGCTCCTCCCTGTTCCA-3′ and 5′-CACACCGACCTTCACCAT-3′. GAPDH was used as the reference gene. All samples were run in triplicate.

### 2.12. 2,2-Diphenyl-1-picrylhydrazyl (DPPH) Radical Scavenging Activity

Free radical scavenging activity was determined by the decolorization of the DPPH radical. In brief, various concentrations of the sample were mixed with a DPPH ethanolic solution (final concentration: 100 μM). Contents were vigorously mixed and incubated at room temperature in the dark for 30 min. The absorbance was measured at 525 nm using a microplate reader (SpectraMax i3, Molecular Devices, CA, USA). Antioxidant activity was expressed as the percentage of DPPH radical elimination and calculated according to the following formula:

[(A_control_ − A_sample_)/A_control_] × 100 (3)

where A_control_ is the absorbance of the control DPPH solution, and A_sample_ is the absorbance of the DPPH solution after addition of the sample. EC_50_ was calculated from the graph of inhibition percentage versus concentration [21]. Analyses were performed using the GraphPad Prism software^®^ (GraphPad Prism Software Inc., version 5.02, La Jolla, CA, USA).

### 2.13. ROS Measurement

C2C12 myoblasts were plated into 4-well chambered slides (0.8 × 10^3^ cells/well, SPL Life Sciences), as well as into 96-well plates (0.5 × 10^2^ cells/well). Seeded cells were allowed to differentiate for 3 days. GdL (3), RosA, and ascorbic acid (AA) (10, 20, and 30 μM) were added with or without palmitic acid (PA) (500 μM). H_2_DCFDA (20 μM, Thermo, D399) was also tested. After incubation for 30 min at 37 °C in the dark, the cells were washed with Hank’s balanced salt solution (HBSS). Chambered slides were fixed with 4% paraformaldehyde for 10 min and washed with Tris-buffered saline (TBS) in the dark. Slide images were immediately captured using a Nikon fluorescence microscope and NIS-Elements BR 5.11 software (Nikon, Tokyo, Japan). Fluorescence of the 96-well plates was measured using a SpectraMax i3 microplate reader (Molecular Devices) set at an excitation wavelength of 480 nm and an emission wavelength of 530 nm. The samples were measured by time kinetics (Figure S9), and 2′,7′-Dichlorofluorescin diacetate (DCF-DA) object mean (fluorescence intensity) of PA-induced ROS data was presented at 30 min of the time points.

### 2.14. Western Blot Analysis

Western blot analysis was performed using a previously described method [22]. Protein expression was assessed via Western blot analysis. Proteins were extracted using radio-immunoprecipitation assay lysis buffer (Millipore, Bedford, MA, USA) containing a protease and phosphatase inhibitor cocktail (Roche, Basel, Switzerland). Cell lysates were then separated via sodium dodecyl sulfate–polyacrylamide gel electrophoresis, and the separated proteins were electrotransferred onto polyvinylidene fluoride (PVDF) membranes. Membranes were blocked in a 3% BSA Tris-buffered saline solution containing 0.05% Tween 20 (TBS-T) for 1 h at room temperature and were incubated with the following diluted primary antibodies: COX-2 (1:2000, Cell Signaling Technologies, Danvers, MA, USA, Catalog No. 12282), super oxide dismutase (SOD) (1:5000, Abcam, Cambridge, MA, Catalog No. 12282), and β-actin (1:1000, Santa Cruz Biotechnology, Santa Cruz, CA, Catalog No. sc47778) in Tris-HCl-based buffer containing 0.1% Tween 20, pH 7.5 (TBS-T buffer). Membranes were incubated with horseradish-conjugated secondary antibody (1:5000, Cell Signaling Technologies) for 1 h at room temperature. After washing with TBS-T, immunoreactive bands were visualized using the Chemiluminescence Western Imaging System (Supernova-Q1800TM, Centronics, Daejeon, Korea). Band intensity was measured using the ImageJ software (National Institute of Health, Bethesda, MD, USA).

### 2.15. Toxicity Test

At 24 h after intravenous injection, all mice were sacrificed under anesthesia with 2.5% isoflurane in supplemental oxygen. Blood was collected from the abdominal aorta in sterile centrifuge tubes and allowed to clot. Serum was separated by centrifuging the samples at 5000 rpm for 15 min after 1.5 h at room temperature and was used for the estimation of serum glutamic oxaloacetic transaminase (GOT) and glutamic pyruvic transaminase (GPT) levels. The levels of GOT and GPT were detected using the ASAN kit (Asan Pharm., Seoul, Korea) based on the Reitman–Frankel method [23]. Moreover, the liver and kidneys were removed, preserved in neutral-buffered formalin, and then processed for paraffin embedding using the standard microtechnique. Three-micron sections of the liver and kidneys stained with hematoxylin and eosin (H&E), and trichrome (Masson) were observed under a microscope for histopathological changes.

### 2.16. Statistical Analysis

Data were evaluated using the one-way analysis of variance with the Tukey’s multiple comparison test. Analyses were performed using GraphPad Prism (GraphPad Prism software Inc., version 5.02, San Diego, CA, USA). Data are expressed as mean ± SD (standard deviation) or standard error of the mean values, and *p* < 0.05 was considered statistically significant.

## 3. Results

### 3.1. Synthesis

**Figure 1:**
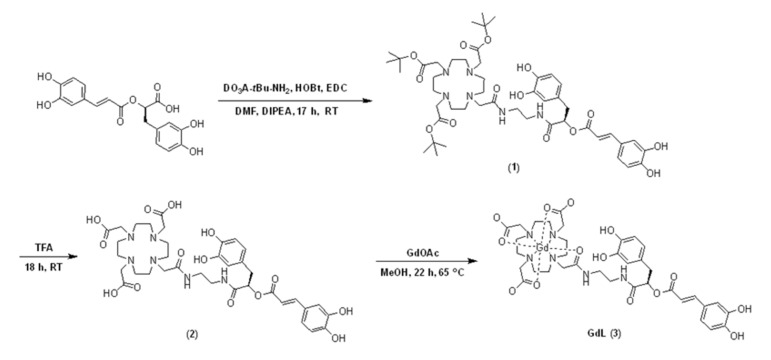
Synthesis of the gadolinium (Gd) complex of the DO3A-rosmarinic acid conjugate.

The bifunctional chelate and its Gd (III) complex, GdL (3), were prepared using Gd-DO3A and RosA, as depicted in Figure 1. The 1-hydroxybenzotriazole hydrate (HOBt)/1-ethyl-3-(3-dimethylaminopropyl) carbodiimide (EDC) amide coupling reaction resulted in the formation of 1. RosA-conjugated DO3A, 2, can be readily obtained by the deprotection of the corresponding tert-butyl ester with TFA. The reaction of Gd chelation using gadolinium acetate resulted in the formation of (Gd(DO3A-RosA)(H_2_O)) as a yellow solid. The formation of 2 and its Gd-complexes, GdL (3), was characterized by ^1^H NMR, ^13^C NMR, high resolution-fast atom bombardment mass (HR-FABMS), UV-vis spectra, and HPLC (Figures S1–S6). The purity of 1, 2, and GdL (3) was determined to be more than 95% by the HPLC spectra (Figure S5).

### 3.2. Physicochemical Characterization

The relative polarity of GdL (3) was estimated using the calculation of the octanol–water partition coefficient (estimated log *P*) values using the ICP technique (Table 1). Estimated log *P* values of GdL (3) and the clinically used gadolinium complex, Gd-BT-DO3A (Gadovist^®^), were −1.75 and 3.13, respectively. Therefore, the lipophilicity of GdL (3) was higher than that of the clinically used gadolinium complex, Gd-BT-DO3A. The two phenol hydroxy groups of RosA may contribute to the lipophilicity of GdL (3). The relaxivity of GdL (3) and Gd-BT-DO3A are also summarized in Table 1. The longitudinal (*r*_1_) and transverse (*r*_2_) relaxivities of GdL (3) in PBS (pH 7.4) were higher than those of Gd-BT-DO3A. A similar result was observed in 0.67 mM HSA solution. This finding is consistent with that of the lipophilicity of Gd-complexes because relaxivity and protein binding correlate strongly with log *p* values [24]. The high lipophilicity of GdL (3) enables strong interaction with HSA.

### 3.3. Kinetic Stability

**Figure 2:**
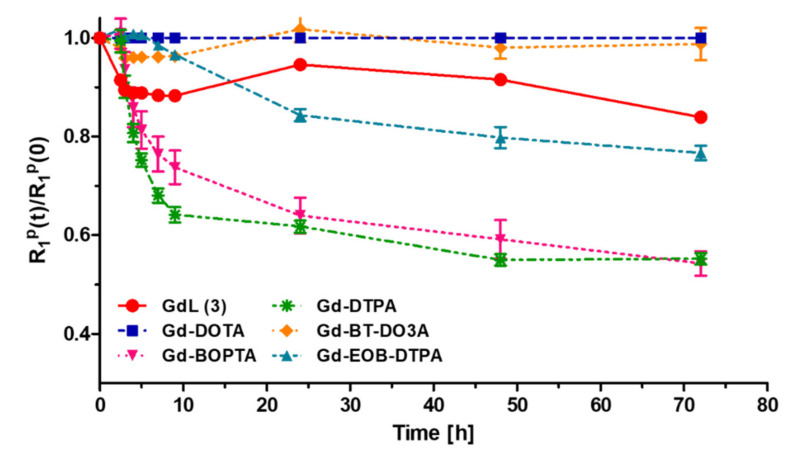
Evaluation of longitudinal relaxation rates *R*_1_*^P^*(t)/*R*_1_*^P^*(0) as a function of time (up to 72 h) for GdL (3) and various magnetic resonance (MR) contrast agents (Gd-BT-DO3A, Gd-DTPA-EOB, Gd-DTPA, Gd-DOTA, and Gd-DTPA-BOPTA) using a 3.0 T MR scanner at 294 K.

Kinetic stability was estimated using a transmetalation study of the complex. Gd can be displaced by Zn^2+^, Cu^2+^, and Ca^2+^ [25]. Zn^2+^ is the best candidate to compete with Gd because of its higher concentration in the blood compared with other competitive ions [19]. Released gadolinium ions might trigger nephrogenic systemic fibrosis (NSF) and Gd accumulation in the brain [26]. The kinetic stability of GdL (3) is comparatively represented by its normalized paramagnetic longitudinal relaxation rates *R*_1_^P^(t)/*R*_1_^P^(0) as a function of time against those of Gd-BT-DO3A (Gadovist^®^), Gd-DOTA (Dotarem^®^), Gd-DTPA-BOPTA (Multihance^®^), Gd-DTPA-EOB (Primovist^®^), and Gd-DTPA (Magnevist^®^) (Figure 2). The value of *R*_1_^P^ at time t is a good estimator of the extent of transmetalation between gadolinium and zinc. Gadolinium-based contrast agents (GBCAs) used for this examination can be classified into two groups depending on the graph pattern: (i) macrocyclic chelates and (ii) linear chelates (Chart S1). As expected, GdL (3) exhibited high values of kinetic inertness that were comparable to that of Gd-BT-DO3A and Gd-DOTA employing the same macrocyclic chelate structure. No significant changes in relaxivity were observed over a period of two days. In contrast, other GBCAs with linear DTPA analogs showed a significant decrease in *R*_1_ during the same period. 

### 3.4. In Vivo Evaluation of Inflammation Targeting

**Figure 3:**
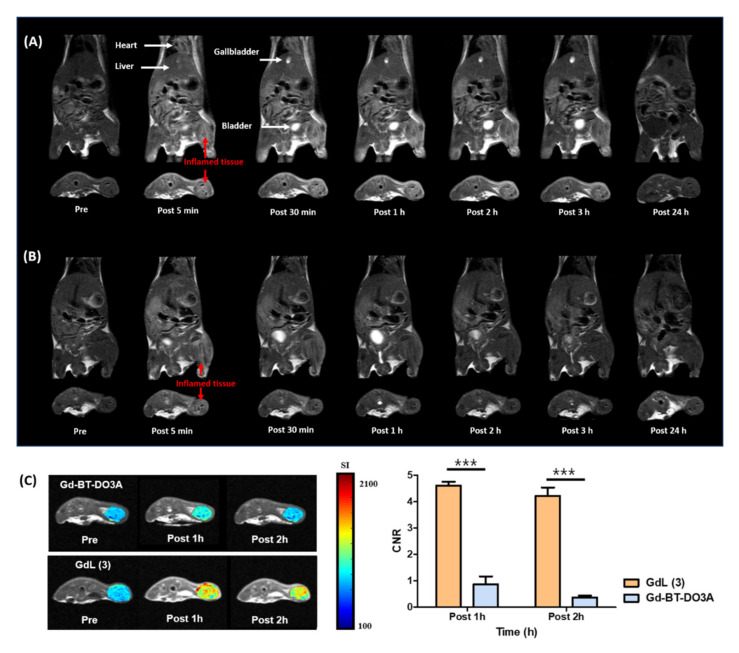
Inflammation targeting of gadolinium and rosmarinic acid (GdL (3)). (**A**,**B**) *T*_1_-weighted MR images of inflammation-induced ICR mice obtained after the intravenous injection of GdL (3) and Gd-BT-DO3A, respectively (0.1 mmol (Gd)/kg, n = 3 for each group). (**C**) Contrast-to-noise ratio (CNR) profiles and in vivo MR axial images of mice bearing inflamed thigh tissues obtained after the injection of GdL (3) and Gd-BT-DO3A. CNR was calculated using Equation (2). *** *p* < 0.001.

The efficacy of GdL (3) to detect inflamed tissues was explored using a mouse inflammation model. Coronal and axial *T*_1_-weighted MR images of whole body and inflamed thigh after the intravenous injection of Gd complexes (0.1 mmol (Gd)/kg) are shown in Figure 3. Gd-BT-DO3A was rapidly excreted in the urine in 2 h, whereas GdL (3) showed high signal intensities in the heart, liver, and gallbladder (Figure 3A,B). A little higher lipophilicity originated from the RosA group in GdL (3), and the *r*_1_ relaxivity of GdL (3) compared to Gd-BT-DO3A can contribute to the high and retained signal intensities of tissues. The biliary and renal excretion of GdL (3) based on the tendency of the CNR are shown in Figure S7. A notable characteristic feature of GdL (3) compared with Gd-BT-DO3A is the substantial enhancement of the signal intensity in the inflamed tissue of the left thigh. The degree of signal enhancement with GdL (3), as measured by CNR, is higher and remains longer than that with Gd-BT-DO3A (Figure 3C). In addition, the signal enhancement lasted for 3 h in the inflamed tissue, indicating inflammation targeting. This could be because the prolonged circulation time due to the strong interaction between GdL (3) and HSA increases the chance to target the inflamed tissue, and the long retention time of GdL (3) at the inflamed tissue, in turn, confers a benefit on the MRI of the inflammation.

### 3.5. In Vitro Cell Toxicity

Prior to performing in vitro studies, the cell viability of GdL (3) and comparative agents, such as RosA, Gd-BT-DO3A, and AA, a positive control for antioxidant activity, were determined using an immortalized mouse myoblast cell line (C2C12) (Figure S8). Cells were incubated with each drug for 24 h. GdL (3) was found to be not cytotoxic over a range of concentrations (0~400 μM). In particular, its cytotoxicity is comparable to that of RosA.

### 3.6. DPPH Free Radical Scavenging Activity

**Figure 4:**
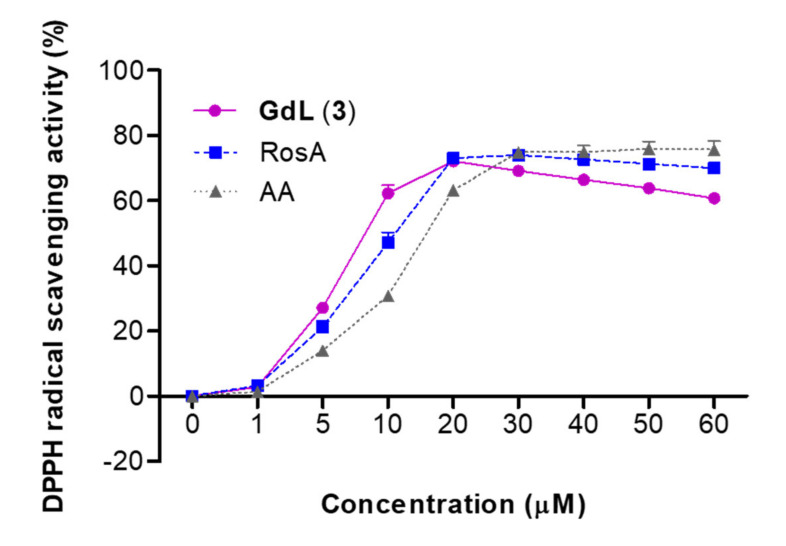
2,2-Diphenyl-1-picrylhydrazyl (DPPH) radical scavenging activity of ascorbic acid (AA), rosmarinic acid (RosA), and GdL (3).

Antioxidant studies were conducted to determine the efficacy of the synthesized GdL (3) as a theranostic agent. A comparison with RosA determined how well the synthesized compound maintained the efficacy of existing polyphenols. DPPH radical scavenging activity experiments were performed using a previously described method [27]. The DPPH radical scavenging activity of GdL (3) was comparable to that of RosA and AA (Figure 4 and Table 2). AA is a well-known antioxidant (vitamin C) and provides a useful comparison with GdL (3) [28]. The scavenging activity against DPPH radicals (half-maximal effective concentration, EC_50_) of GdL (3) was 10.51 μM within a concentration range of 1–30 μM. This activity was significantly greater than that of AA (15.51 μM) and was comparable to that of RosA (11.73 μM).

### 3.7. GdL (3) as an Intracellular ROS Scavenger

**Figure 5:**
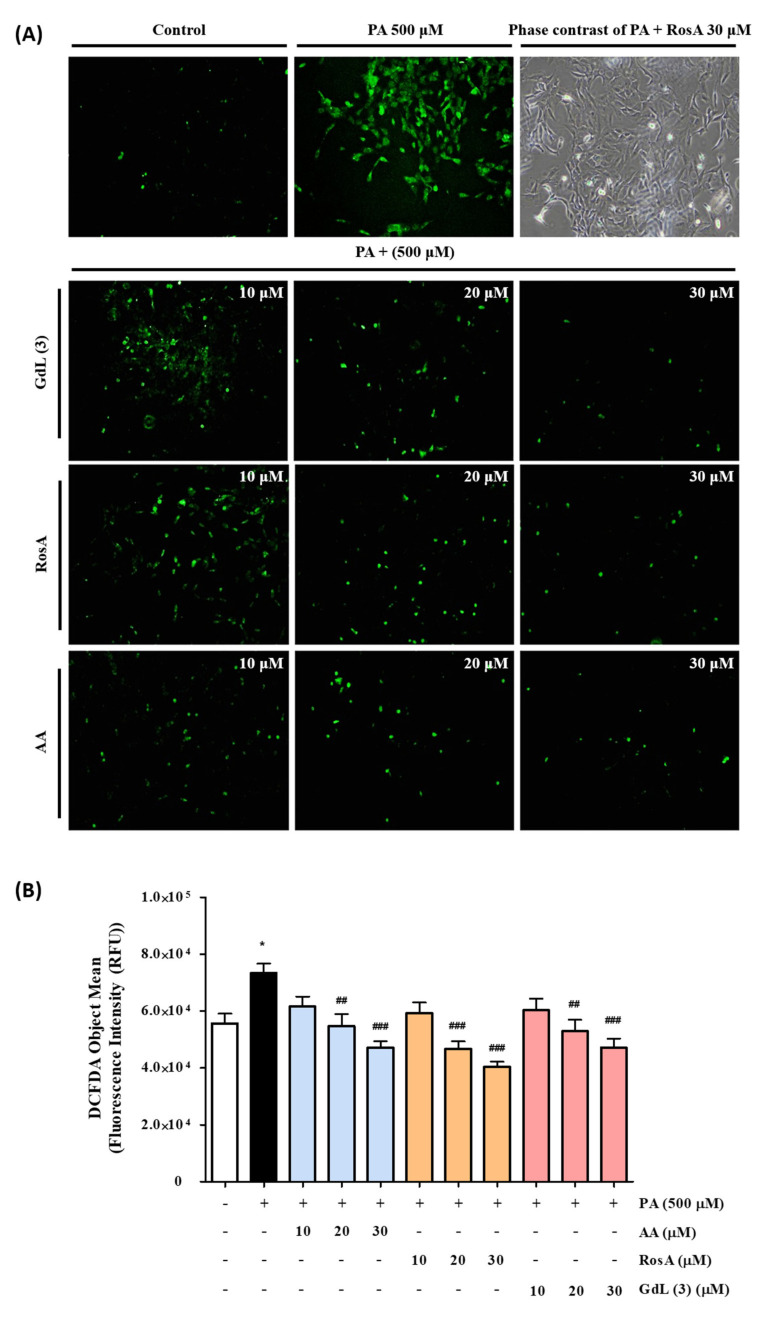
Determination of cellular palmitic acid (PA)-induced reactive oxygen species (ROS) by the DCF-DA assay. (**A**) Fluorescence microscopic images of the control and AA, RosA, and GdL (3) treatments. (**B**) DCF-DA object mean (fluorescence intensity) of PA-induced ROS. * *p* < 0.05, significant difference from the control. *^##^ p* < 0.01 and *^###^ p* < 0.001, significant difference from PA (n = 3 per group).

To confirm the antioxidant effect of GdL (3), intracellular ROS scavenging capacity was evaluated using the 2′,7′-dichlorodihydrofluorescein diacetate (DCFH-DA) assay, a widely used technique to directly measure the redox state of cells. DCFH-DA is converted to the fluorescent compound DCF following ROS-mediated oxidation [29]. Figure 5 shows the fluorescence microscopy images of the increased DCF content caused by PA-induced ROS in C2C12 cells and a decreased DCF content after the treatment of cells with GdL (3), AA, and RosA at concentrations of 10–30 μM. Phase-contrast images showed that the generation of ROS decreases in the presence of GdL (3) without a concomitant decrease in the cell number. All three compounds reduced ROS in a dose-dependent manner. The ROS scavenging efficacy of GdL (3) was similar to that of AA and RosA within the margin of error. The antioxidant effect of RosA was not reduced by structural modification as a Gd complex.

### 3.8. In Vitro and In Vivo SOD Activity of GdL (3) as an Antioxidant

**Figure 6:**
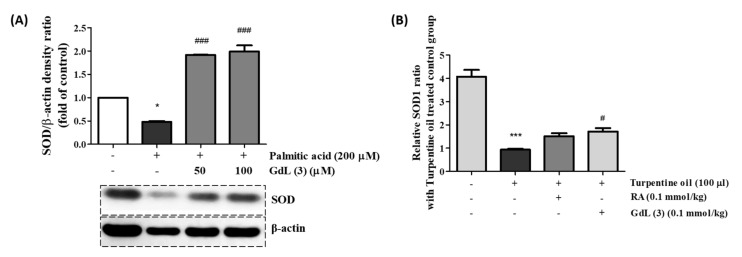
The efficacy of the GdL (3) treatment on the super oxide dismutase (SOD) expression in (**A**) PA-induced C2C12 cells and (**B**) turpentine oil-induced inflammatory tissue. * *p* < 0.05 and *** *p* < 0.001, significant difference from the control. *^###^ p* < 0.001, significant difference from the group of PA-induced cells (n = 4) and *^#^ p* < 0.01, significant difference from the group of only turpentine oil-treated mice (n = 6).

SOD is a widely known antioxidant enzyme present in natural cellular defenses. SOD converts superoxide radicals (O_2_^●^^−^) to hydrogen peroxide (H_2_O_2_), which is then converted to H_2_O by catalase. A decrease in SOD activity causes an excess of O_2_^●^^−^ and H_2_O_2_, with a concomitant increase in oxidative stress. Therefore, SOD activity is an important indicator of the antioxidant status [30]. In vitro and in vivo SOD activity studies were performed to determine the antioxidant activity of GdL (3). First, PA-induced C2C12 cells were treated with GdL (3). The concentration of PA for this study, 200 μM, was sufficient to downregulate the SOD protein expression (Figure S10A). In the presence of 100 μM GdL (3), the SOD activity was more than two-fold that of the PA-treated group (Figure 6A). A parallel result was observed in a turpentine oil-induced inflammatory mice model. At 24 h after the injection of GdL (3), the SOD activity was increased by approximately 1.8-fold compared with the nontreated group. Therefore, GdL (3) exhibits an antioxidant role by scavenging ROS.

### 3.9. Anti-Inflammatory Activity of GdL (3)

**Figure 7:**
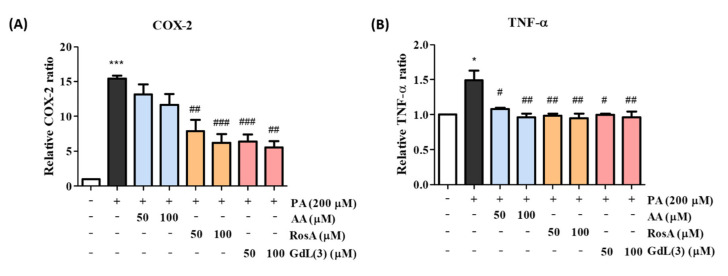
Anti-inflammatory effects of RosA and GdL (3) on PA-induced inflammation in C2C12 cells. The mRNA expression levels of (**A**) cyclooxygenase-2 (COX-2) and (**B**) tumor necrosis factor (TNF)-α according to drug concentration. * *p* < 0.05, *** *p* < 0.001 significant difference from the control. ^#^ *p* < 0.05, ^##^ *p* < 0.01, and ^###^ *p* < 0.001, significant difference from PA. This experiment was shown by averaging the results of three independent experiments.

The mRNA expression levels of COX-2 and TNF-α were measured via real-time PCR to confirm the anti-inflammatory activity of GdL (3). COX-2 expression is upregulated during the inflammation process, resulting in the induction of prostaglandins involved in inflammatory reactions and cell proliferation [31]. TNF-α is also a key inflammatory cytokine that is secreted by activated macrophages. In addition, it is known to induce COX-2 production [32]. Therefore, the suppression of COX-2 and TNF-α is an important activity exhibited by anti-inflammatory agents. PA at a concentration of 200 μM, which sufficiently stimulated COX-2 in a preliminary experiment, was used to induce the mRNA expression of COX-2 and TNF-α for anti-inflammatory studies (Figure S10B). mRNA expression was observed for COX-2 and TNF-α in PA-induced C2C12 cells after treatment with two concentrations (50 and 100 μM) of AA, RosA, and GdL (3) using real-time PCR (Figure 7). COX-2 expression decreased after treatment with all compounds, demonstrating their anti-inflammatory properties. Cells treated with 100-μM GdL (3) showed significant COX-2 suppression compared with those treated with 100-μM AA and slightly better activity compared with those treated with 100-μM RosA (Figure 7A). In contrast, TNF-α expression was downregulated after treatment with all compounds (Figure 7B).

**Figure 8:**
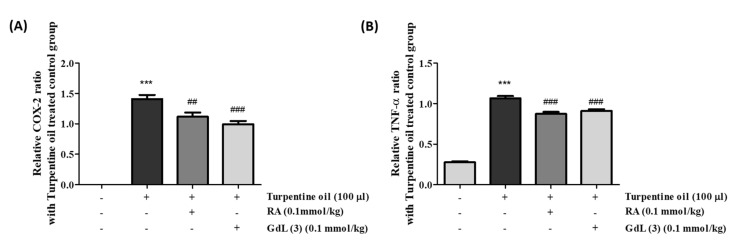
Anti-inflammatory effects of RosA and GdL (3) on turpentine oil-induced inflammation (ICR mice). The mRNA expression levels of (**A**) COX-2 and (**B**) TNF-α according to drug concentration. *** *p* < 0.05, significant difference from the control. *^##^ p* < 0.01 and *^###^ p* < 0.001, significant difference from PA (n = 3–6 per group).

Based on the in vitro anti-inflammatory activity, GdL (3) was intravenously administered to mice with turpentine oil-induced inflammation. The dose of 100-μM turpentine oil was decided to induce COX-2 and TNF-α compared to the dose of 150 μM, because there are no significant differences (Figure S11). After 24 h, the inflamed thigh tissue was obtained and analyzed using real-time PCR. The results of these in vivo anti-inflammatory experiments are similar to those of the in vitro assays (Figure 8). The anti-inflammatory activity of GdL (3) for the suppression of COX-2 and TNF-α expression was comparable to that of RosA.

### 3.10. Toxicity of GdL (3)

**Figure 9:**
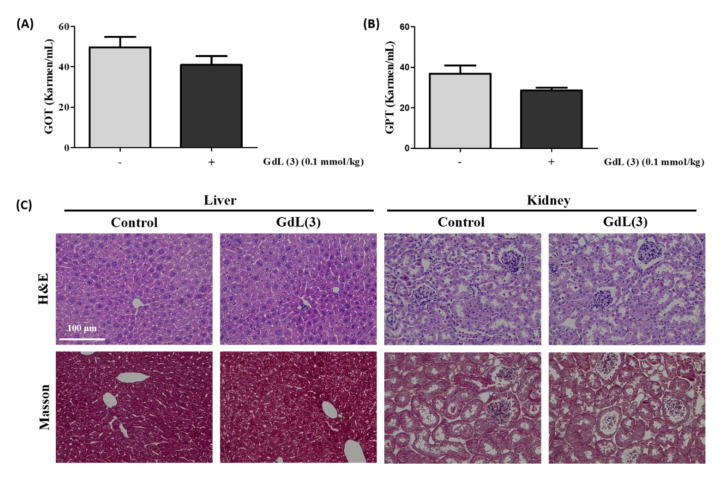
Toxicity data of GdL (3). (**A**) Glutamic oxaloacetic transaminase (GOT) and (**B**) glutamic pyruvic transaminase (GPT) data of GdL (3). (**C**) Micrographs of hematoxylin and eosin (H&E)- and Masson-stained liver and kidney tissue slices before and after 24 h of GdL (3) treatment (n = 3 per each group). Scale bar = 100 µm.

GOT and GPT are known indicators of tissue damage. Their levels were measured at 24 h after injection to determine the toxicity of GdL (3). Regarding the activity of these enzymes, no effects on the GOT and GPT activities were observed as a consequence of the injection of GdL (3) compared with the nontreated group of mice (Figure 9A,B). In addition, a comprehensive histological study of the liver and kidney tissue section was performed to investigate whether the GdL (3) treatment causes any impairment. H&E and Masson-staining studies of tissues did not present any apparent injury in the cellular structures after a 24-h injection of GdL (3) compared with the tissues without GdL (3) injection. There were no significant changes in morphology up to 24 h after the injection of GdL (3). These results imply the in vivo stability of GdL (3) as a theranostic agent.

## 4. Discussion

Oxidative stress, referred to as excessive ROS in cells and tissues [3], is associated with various pathogenesis, including cardiovascular disease [33,34], diabetes [35], Alzheimer’s disease [36], inflammatory disease [33], carcinogenesis [37], and neurodegenerative disease [38]. Inflammation can also be considered a prime factor of many diseases and is linked to the development of various diseases and is triggered by oxidative stress [39]. Therefore, antioxidants are used as drug for inflammatory diseases. They react with free radicals to neutralize and prevent or reduce damage to the body. There has recently been a remarkable growth in the field of theranostics for inflammatory diseases [40]. In particular, polyphenols, including phenolic acids and flavonoids, are good candidates for use as theranostic agents by enabling theranostic technology transfer to inflammation research. RosA is one such phenolic compound. It contributes to its anti-inflammatory activity by interrupting the ROS–inflammation relationship, such as ROS scavenging, metal chelation, ROS suppression, and ROS detoxification [41]. For example, polyphenols such as quercetin and curcumin can chelate metal ions to interrupt the OH^●^ formation from H_2_O_2_ [42,43]. In addition, apocynin and reservatol can inhibit nitrogen oxide (NOX), resulting in the decrease in the generation of O_2_^●^ during inflammation [44,45]. Taken together, the ROS scavenging activities of polyphenols are attributed to their specific chemical structures [46,47]. The caffeic acid moiety of RosA is oxidized and can consequently reduce the formation of oxidative damage products by the direct neutralization of ROS [48]. In our study, GdL (3) demonstrated excellent radical scavenging activity, even though the Gd complex was conjugated (Figure 4 and Table 2), thereby illustrating that its ROS scavenging ability was also excellent (Figure 5). However, ROS are important signaling molecules to maintain a normal physiological function, and their regulation in the disease is complicated. Furthermore, it is important to investigate possible ROS modulations by theranostics. Therefore, further study about the regulation effect of GdL (3) as an antioxidant in various physiological conditions is necessary.

RosA exhibits various biological properties, including anticancer [49], anti-inflammatory [50,51], antidepressant [52], antioxidant [53], antineuropathy [54], and hepatoprotective effects [55]. Many in vitro and in vivo studies have reported the anti-inflammatory effects of RosA in various inflammatory diseases, including arthritis, colitis, atopic dermatitis, asthma, allergic rhinitis, periodontal diseases, and acute pancreatitis and mastitis [56]. The anti-inflammatory effect in the sciatic nerve chronic constriction injury (CCI)-induced neuropathic pain model [51] and anticancer effect in the hepatocellular carcinoma (HCC) animal model [57] are reported to be caused by RosA, which regulates the expression of TNF-α, COX-2, IL-1β, and p65. Our study also demonstrated that GdL (3) regulates the expression of the inflammatory factors COX-2 and TNF-α in the PA-induced in vitro inflammation model (Figure 7) and turpentine oil-induced in vivo inflammation model (Figure 8). In addition, proteins associated with enzyme-mediated antioxidant mechanisms include SOD, glutathione peroxidase, and catalase [58]. These enzymes are primarily activated in response to ROS. We observed that, although GdL (3) has weak activity in the in vitro and in vivo inflammatory models, it statistically significantly restores the expression of SOD (Figure 6).

In the present study, we demonstrated a new type of *T*_1_ MRI contrast agent as a theranostic agent with antioxidant and anti-inflammatory activities promoted by the ROS scavenging of RosA (Figure 4). In structural consideration, a study regarding RosA has shown that the carboxylic group of RosA demonstrates limited cell membranes penetration and intracellular action, whereas the amide derivatives of RosA are effective in preventing H_2_O_2_-induced DNA damage and exhibit less cytotoxicity [59]. Based on this, GdL (3) possessing amide bonding between the Gd complex and RosA (Figure 1) can readily diffuse via the cell membrane, boosting the inflammation-targeting effect and prolonging MR enhancement in the inflamed tissue. These results suggest that the therapeutic effect of GdL (3) is attributed to conjugated RosA. Further studies regarding the antioxidant and anti-inflammatory mechanisms of the new theranostic agent, GdL (3), should be considered. We confirmed that GdL (3) exhibits no toxicity to liver tissue and no cytotoxicity to the kidneys (Figure 9). Therefore, the RosA-conjugated Gd complex, GdL (3), facilitates specific antioxidant and anti-inflammatory therapies based on a single molecule for MR theranosis.

## 5. Conclusions

A bifunctional complex of RosA conjugated to Gd, GdL (3), was designed and synthesized as a new single-molecule antioxidant/anti-inflammatory theranostic agent. Its *r*_1_ relaxivity is higher than that of Gd-BT-DO3A, and its kinetic inertness is similar to that of structurally related cyclic gadolinium chelates. In MRI, the inflamed tissues of mice administered with GdL (3) were specifically enhanced and maintained for 3 h, demonstrating inflammatory targeting. Its antioxidant and anti-inflammatory effects were established by ROS scavenging and the suppression of inflammatory factors COX-2 and TNF-α. An evolved modification of the RosA structure displayed properties of a useful theranostic agent possessing a MR diagnostic ability and antioxidant/anti-inflammatory activities.

## Figures and Tables

**Table 1:** Relaxivity and octanol–water partition coefficients of GdL (3) and Gd-BT-DO3A in PBS and HSA using a 3.0 T MR scanner at 294 K.

MRI Contrast Agents	*r*_1_ (mM^−1^S^−1^)		*r*_2_ (mM^−1^S^−1^)		log *P*_oct/wat_
	PBS	HSA	PBS	HSA	
GdL (3)	5.75 ± 0.23	6.01 ± 0.26	7.32 ± 0.04	13.94 ± 0.20	−1.75
Gd-BT-DO3A	3.74 ± 0.18	4.10 ± 0.21	5.09 ± 0.04	6.07 ± 0.07	−3.13

Phosphate-buffered saline (PBS) (pH 7.4) and human serum albumin (HSA) (0.67 mM) were used. Values are expressed as mean ± SD (n = 3).

**Table 2:** 2,2-Diphenyl-1-picrylhydrazyl (DPPH) radical scavenging activity of ascorbic acid (AA), rosmarinic acid (RosA), and GdL (3).

Chemicals (μM)	DPPH Scavenging (%)					EC_50_ Value (μM)	R^2^
	1	5	10	20	30		
GdL (3)	2.87 ± 2.76	27.19 ± 5.57 ***	62.29 ± 8.24 ***	72.18 ± 4.74 ***	69.20 ± 3.94	10.51	0.8361
RosA	3.13 ± 3.14	21.14 ± 6.30 *	47.02 ± 10.9 ***	73.05 ± 3.31 ***	73.96 ± 4.52	11.73	0.9113
AA	1.34 ± 1.53	13.99 ± 1.60	30.70 ± 1.31	63.13 ± 3.26	74.83 ± 4.95	15.51	0.9588

Values are expressed as mean ± SD (n = 3). * *p* < 0.05 and *** *p* < 0.001 significant differences from the standard.

## Supplementary Materials

The following are available online at https://www.mdpi.com/2076-3921/9/8/744/s1, Chart S1: Clinically used MRI contrast agent. Figures S1 and S2: HR-FAB mass of the synthesized compounds **1**, **2**, and GdL (3). Figures S3 and S4: ^1^H NMR spectroscopy data of the compounds **1** and **2**. Figure S5: HPLC data for the purity of the compounds **1**, **2**, and GdL (3). Figure S6: Absorption and emission spectra of GdL (3). Figure S7: Biodistribution data of GdL (3) and Gd-BT-DO3A. Figure S8: Cell viability. Figure S9: DCF-DA object mean of PA-induced ROS. Figure S10: Western blot expression pattern data of SOD and COX-2. Figure S11: Expression of COX-2 and TNF-α in mice with turpentine oil injection.

## Abbreviations

RosA	rosmarinic acid
(TNF)-α	tumor necrosis factor-α
(IL)-1β	interleukin-1 β
VEGF	vascular endothelial growth factor
ROS	reactive oxygen species
COX-2	cyclooxygenase-2
NF-κB	nuclear transcription factor-κB
MOFs	metal-organic frameworks
MRI	magnetic resonance imaging
HSA	human serum albumin
NSF	nephrogenic systemic fibrosis
GBCAs	gadolinium-based contrast agents
CNR	contrast-to-noise ratio
AA	ascorbic acid
SOD	superoxide dismutase
