# Supplementary material for: Gd-Complex of a Rosmarinic Acid Conjugate as an Anti-Inflammatory Theranostic Agent via Reactive Oxygen Species Scavenging
Source: Antioxidants (Basel). 2020 Aug 13;9(8):744. doi: 10.3390/antiox9080744 (PMC7464237; doi:10.3390/antiox9080744)
Supplement: Supplementary file 1 [file antioxidants-09-00744-s001.pdf]

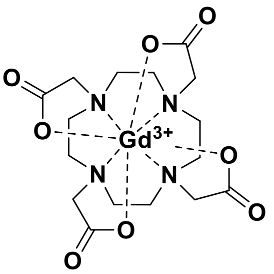

Gd-DOTA

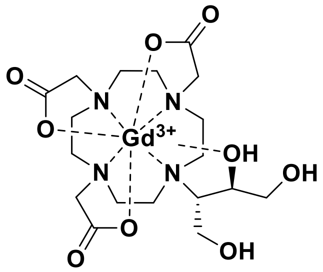

Gd-BT-DO3A

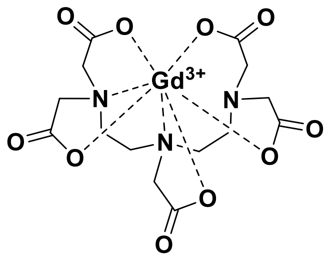

Gd-DTPA

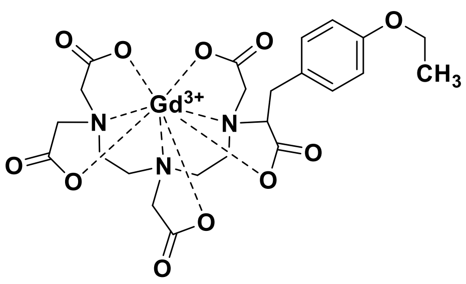

Gd-EOB-DPTA

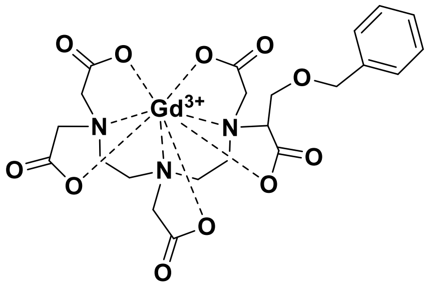

Gd-BOPTA

Chart S1. Clinically used MRI contrast agents.

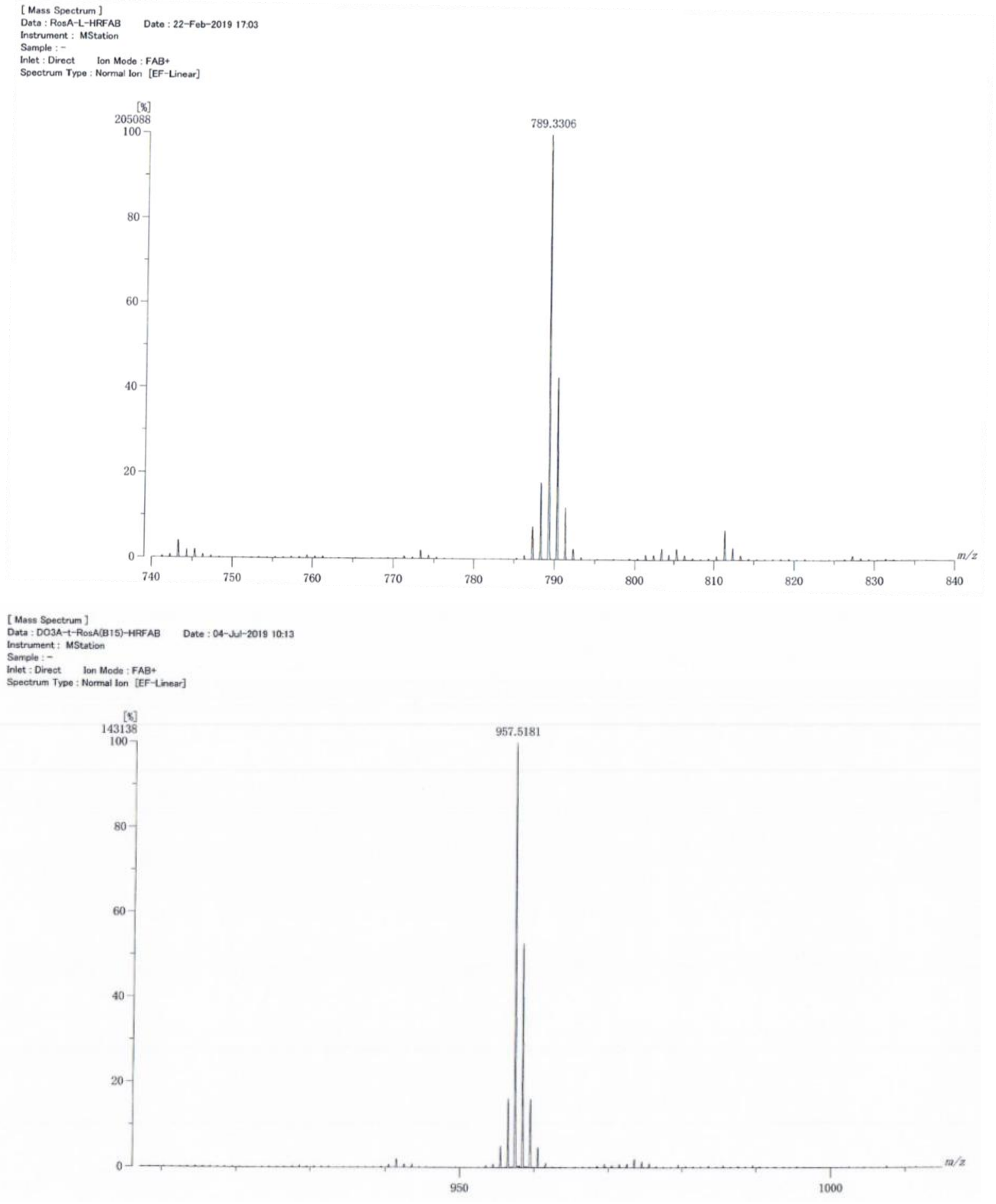

Figure 1. HR-FAB mass spectroscopy data of (1) (Top) and (2) (Bottom).

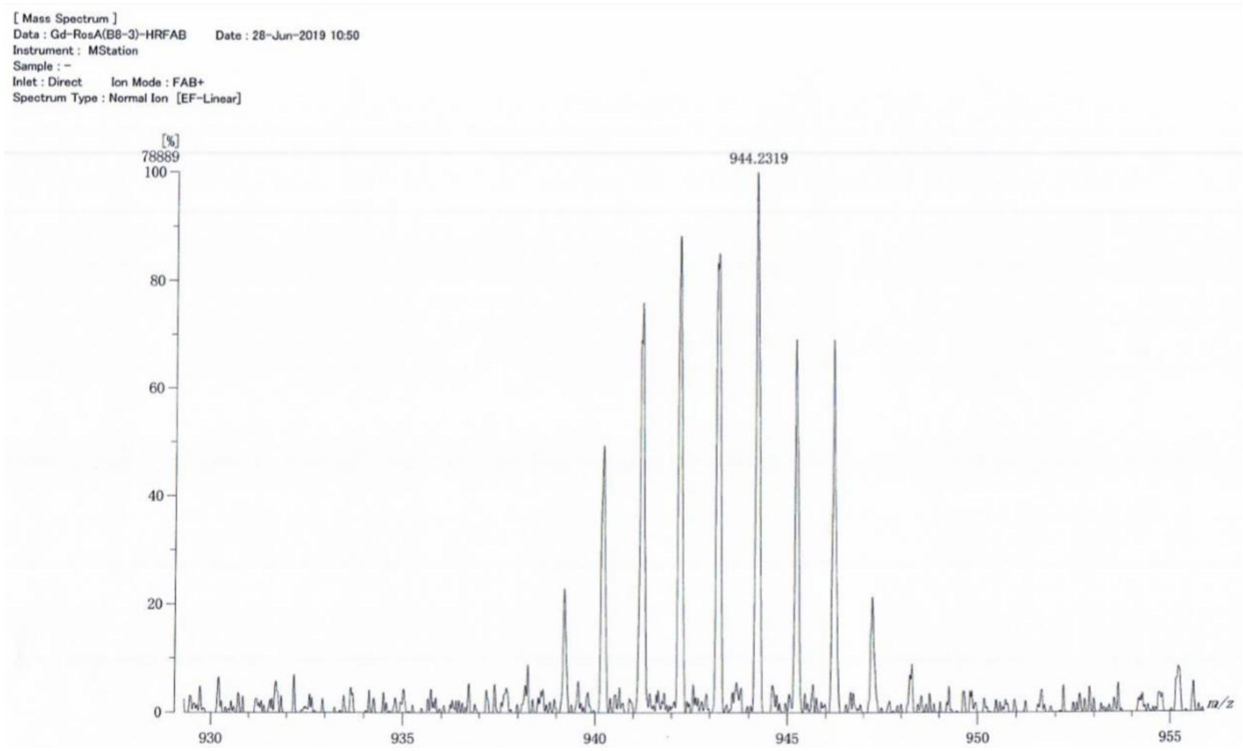

Figure 2. HR-FAB mass spectroscopy data of GdL (3).

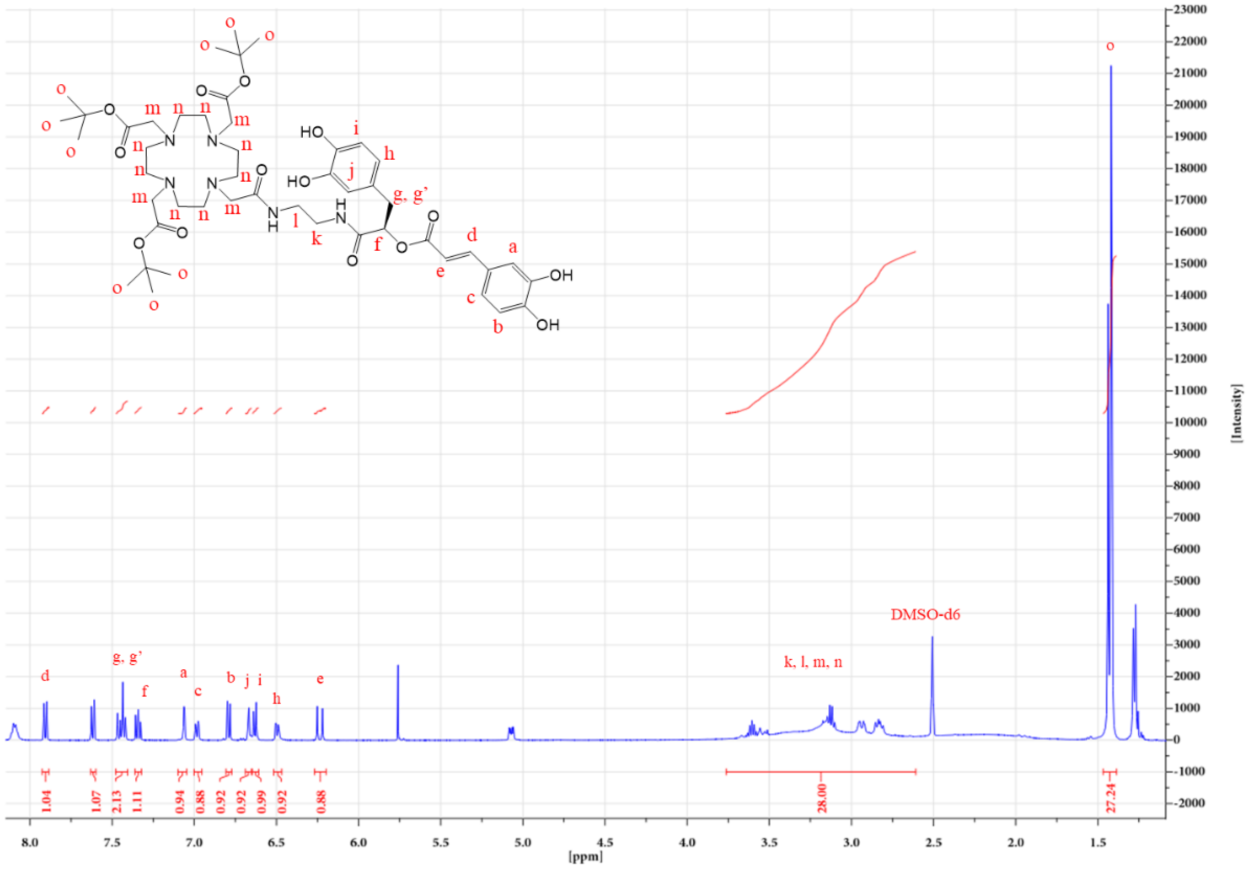

Figure 3. ^{1}H NMR spectroscopy data of (**1**).

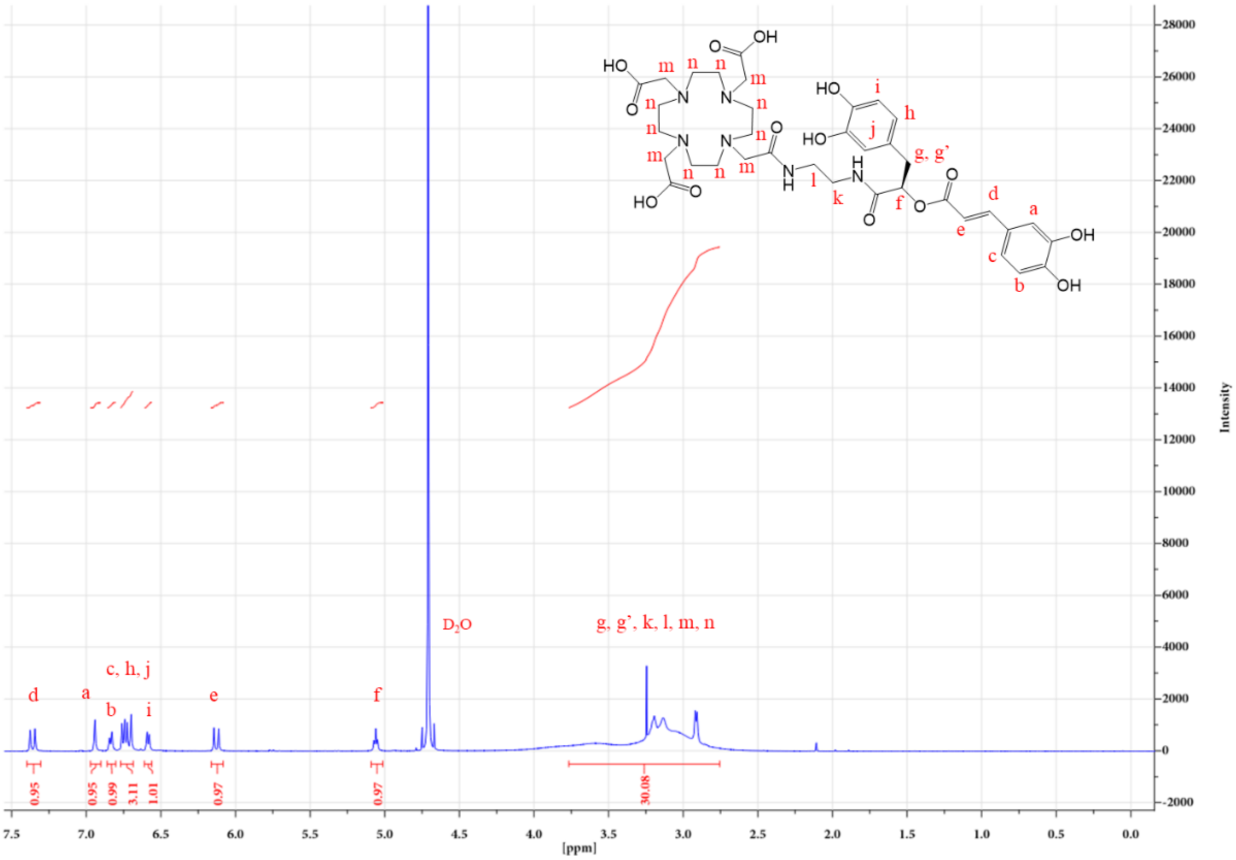

Figure 4. ^{1}H NMR spectroscopy data of (2).

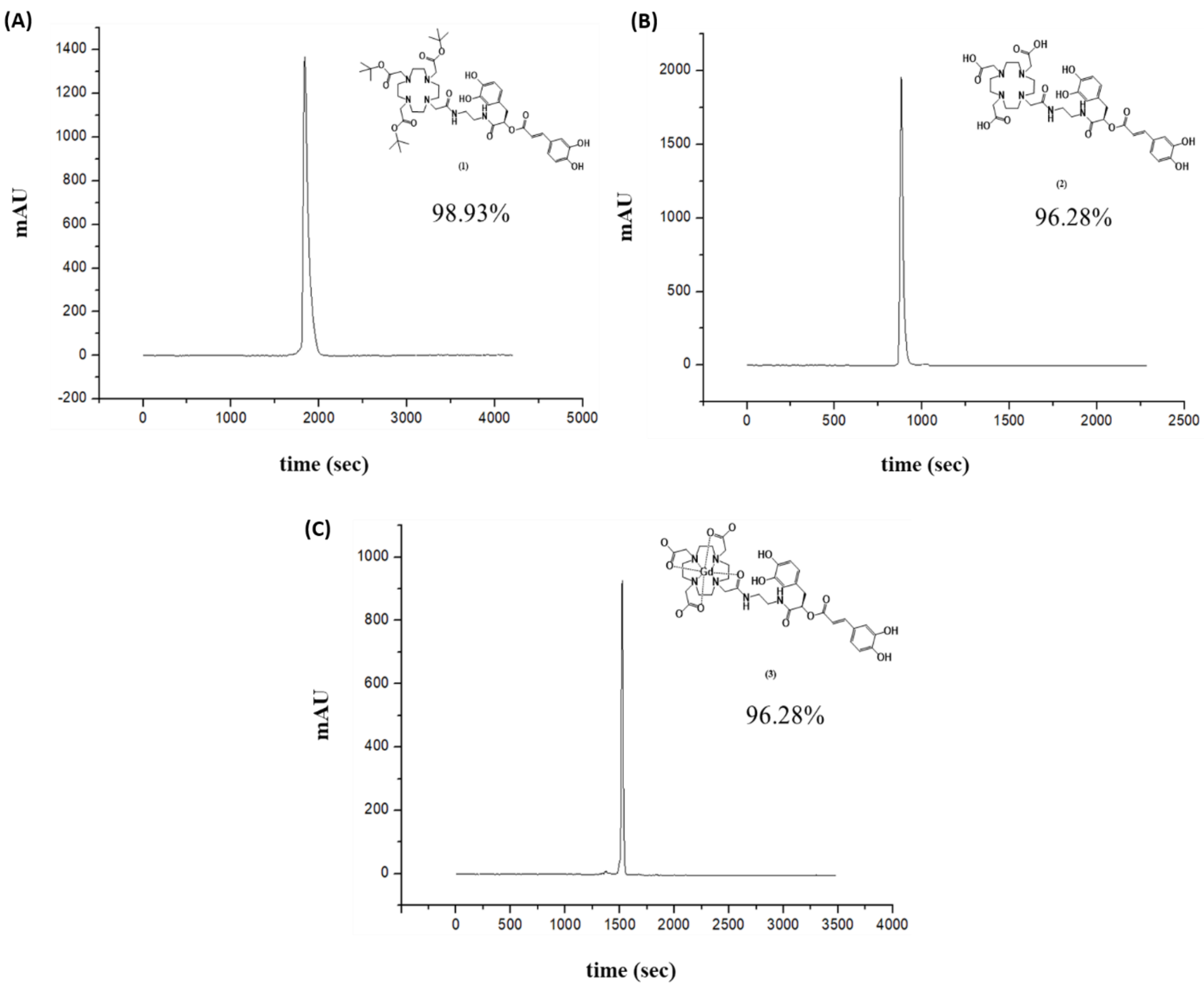

Figure 5. Peak purity in Prep-HPLC of (1) at 365 nm, (2) and GdL (3) at 330 nm detection.

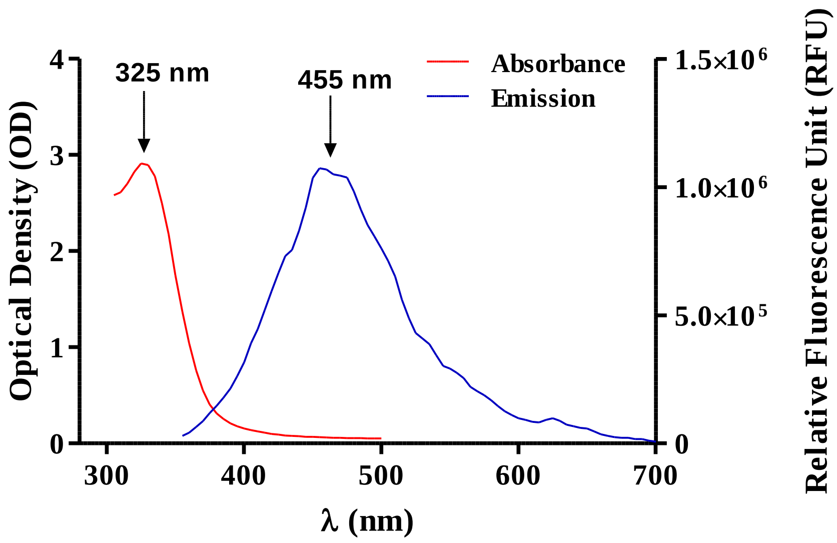

Figure 6. Absorption (Red) and Emission (Blue) spectra of GdL (3).

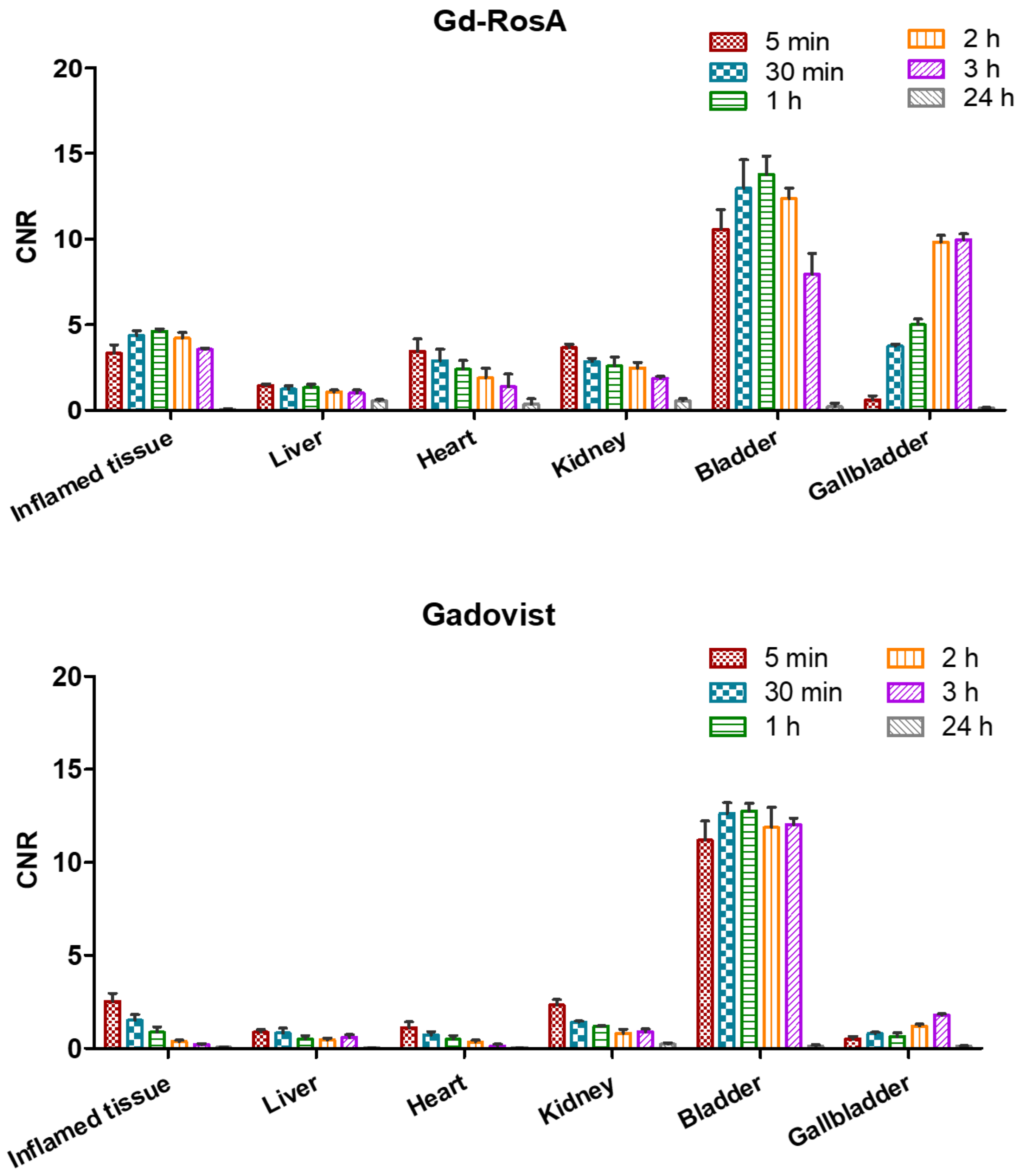

Figure 7. Biodistribution of **GdL** (**3**) and Gd-BT-DO3A (Gadovist®) in various tissues (each group n = 3).

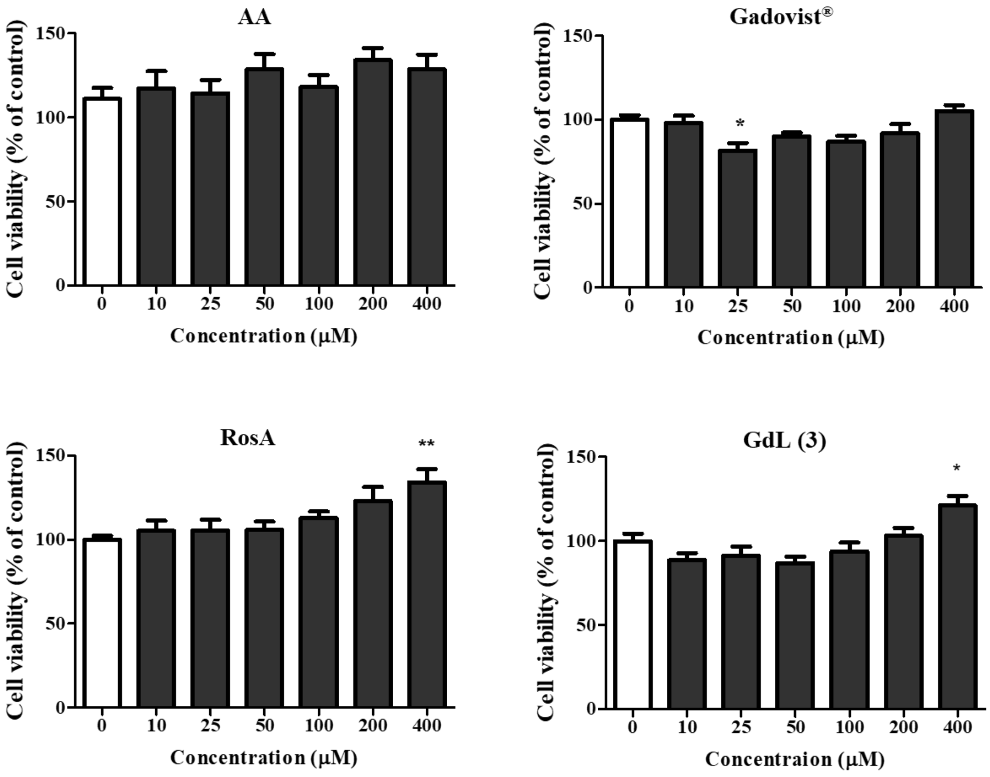

Figure 8. Cell viability of immortalized mouse myoblast cell line (C2C12) in various AA, Gd-BT-DO3A, RosA and **GdL** (**3**) concentrations.

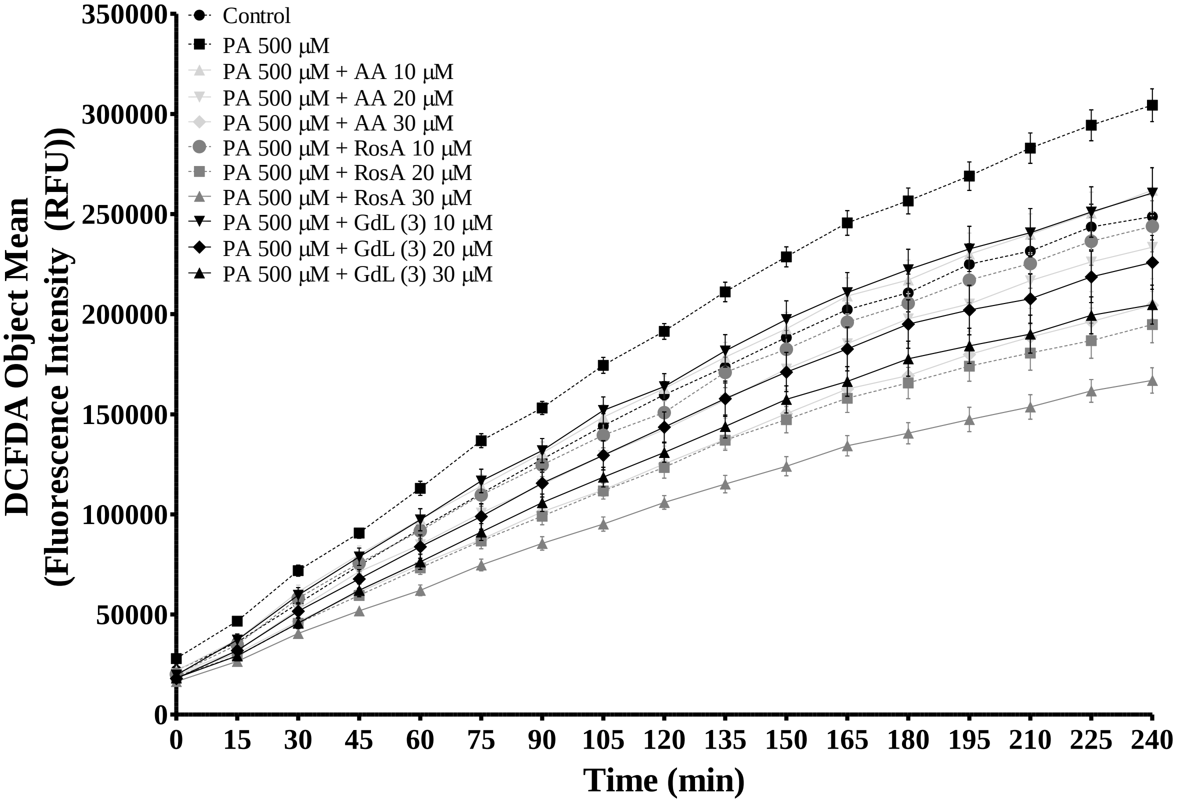

Figure 9. DCFDA object mean (fluorescence intensity) of PA-induced ROS.

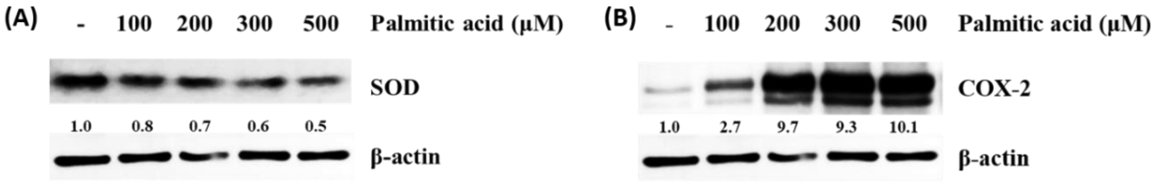

Figure 10. The expression pattern of (A) SOD and (B) COX-2 according to various concentrations (100, 200, 300, and 500 μM) of palmitic acid. (n = 3 per each).

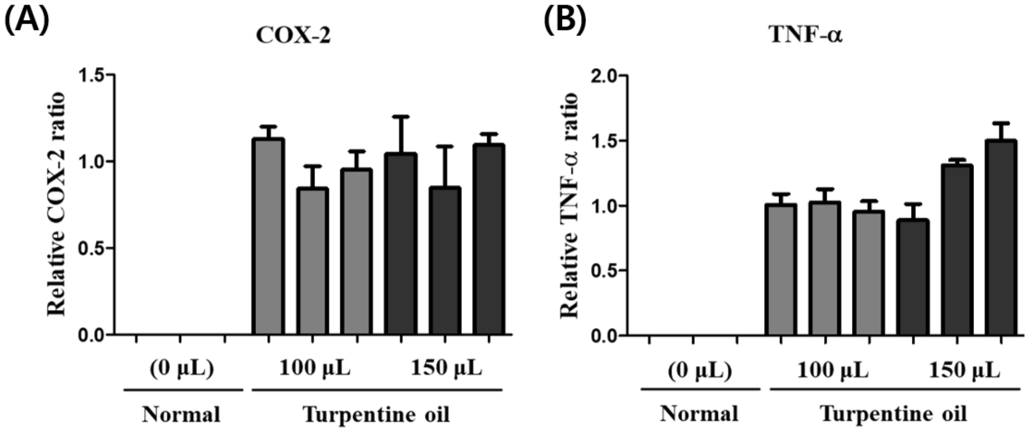

Figure S11. Expression of COX-2 and TNF-α in mice with turpentine oil injection dose. (A) COX-2 expression level according to turpentine oil injection dose. (B) TNF-α expression level according to turpentine oil injection dose.
